# Reply to “Letter to the Editor for the Review Paper: The association between blood pressure variability with dementia and cognitive function: A systematic review and meta‐analysis”

**DOI:** 10.1111/jch.14357

**Published:** 2021-08-27

**Authors:** Tzu‐Jung Chiu, Jiunn‐Tyng Yeh, Hao‐Min Cheng

**Affiliations:** ^1^ Department of Medicine National Yang Ming Chiao Tung University College of Medicine Taipei Taiwan; ^2^ Institute of Public Health National Yang Ming Chiao Tung University College of Medicine Taipei Taiwan; ^3^ Center for Evidence‐based Medicine Taipei Veterans General Hospital Taipei Taiwan; ^4^ Department of Medical Education Taipei Veterans General Hospital Taipei Taiwan; ^5^ Institute of Health and Welfare Policy National Yang Ming Chiao Tung University College of Medicine Taipei Taiwan

To the Editor, we appreciate the comments from Tully and de Heus and would like to respond to their concerns. As widely recognized, systematic review and meta‐analysis, due to its timely summary and analysis through comprehensive search for the best available research, is an useful research strategy to summarize the totality of evidence.^1^ The *a priori* registration of protocols for a systematic review and meta‐analysis is recommended by the reporting guideline of PRISMA (Preferred Reporting Items for Systematic Reviews and Meta‐Analyses). Though not mandatory, the protocol directs, yet not limits, the study of the research topic, and ensures the additive value of each meta‐analysis with the latest evidence. According to the developers of the PROSPERO, protocol development is iterative and it is important to record and amend the registry during major changes in methods that may potentially result in biases.^2^ During the process of this recently published systematic review and meta‐analysis,^3^ we found that the current literature is insufficient to answer our original research question, hence we extended the scope and conducted more subgroup analysis and dose‐response meta‐analysis. The limitation of our study and the necessity of conducting more clinical studies have been clearly indicated in the discussion section of our manuscript. Apart from that, we have submitted to PROSPERO an update and amendment of our protocol elaborating the changes of methods, yet due to the COVID‐19 pandemic, the new version has not been publicized. We are hoping to clarify the confusion raised by the discrepancy between the yet‐to‐be‐updated protocol and our manuscript; however, we do not think the amendment and update of the protocol should prevent the research community from receiving the timely evidence synthesis for this clinically important issue with huge global impact.

We were aware of the PROSPERO protocol submitted by the VARIABLE BRAIN consortium registered in December, 2017 with the anticipated completion in December 31, 2018; however, there was no further update after the announcement the start of the formal screening in August 28, 2018, which made it difficult to judge if the project was ongoing.^4^ Nevertheless, we specifically avoided tackling the identical clinical question and carried out the study from different perspectives, including the comparisons of the prognostic values of different indices or follow‐up time of BPV, as well as a dose‐response meta‐analysis by summarizing the most up‐to‐date studies. Indeed, due to the scarcity of available studies, our study had limitations, which has been described in the discussion of our paper. Hence, we are pleased to see the preliminary results of the meta‐analysis from the VARIABLE BRAIN consortium with a bigger effect size and more comprehensive subgroup analysis,^5^ which will hopefully provide a deeper insight into the prognostic role of the blood pressure variability for incident dementia. We look forward to reading this long‐awaited publication with the meta‐analysis results from this esteemed consortium.

## References

[1] Tawfik GM , Giang HTN , Ghozy S , et al. Protocol registration issues of systematic review and meta‐analysis studies: a survey of global researchers. BMC Med Res Methodol. 2020;20(1):213. 32842968 10.1186/s12874-020-01094-9 PMC7448304

[2] Booth A , Clarke M , Dooley G , et al. The nuts and bolts of PROSPERO: an international prospective register of systematic reviews. Syst Rev. 2012;1(2). 10.1186/2046-4053-1-2 PMC3348673 22587842

[3] Chiu T‐J , Yeh J‐T , Huang C‐J , et al. Blood pressure variability and cognitive dysfunction: a systematic review and meta‐analysis of longitudinal cohort studies. J Clin Hypertens. 2021. 10.1111/jch.14310 PMC8678719 34153171

[4] Tully P , Turnbull D , Cosh S , The association between blood pressure variability (BPV) with dementia and cognitive function: a systematic review and meta‐analysis; https://www.crd.york.ac.uk/prospero/display_record.php?ID=CRD42017081977, 2021. 10.1186/s13643-018-0811-9 PMC6190539 30322404

[5] De Heus R , Tully P , Consortium NVB . The association of blood pressure variability with dementia and cognitive impairment: a systematic review and meta‐analysis. J Hypertens. 2021;39:e182. 10.1161/HYPERTENSIONAHA.121.17797 PMC8516811 34538105

